# Garbage Management: An Important Risk Factor for HPAI-Virus Infection in Commercial Poultry Flocks

**DOI:** 10.3389/fvets.2018.00005

**Published:** 2018-01-26

**Authors:** Emily Walz, Eric Linskens, Jamie Umber, Marie Rene Culhane, David Halvorson, Francesca Contadini, Carol Cardona

**Affiliations:** ^1^Department of Veterinary and Biomedical Sciences, University of Minnesota, St. Paul, MN, United States; ^2^Department of Veterinary Population Medicine, University of Minnesota, St. Paul, MN, United States; ^3^Center for Animal Health and Food Safety, University of Minnesota, St. Paul, MN, United States

**Keywords:** United States, poultry, farms, chickens, turkeys, risk, waste disposal facilities, highly pathogenic avian influenza

## Abstract

Garbage management represents a potential pathway of HPAI-virus infection for commercial poultry operations as multiple poultry premises may share a common trash collection service provider, trash collection site (e.g., shared dumpster for multiple premises) or disposal site (e.g., landfill). The types of potentially infectious or contaminated material disposed of in the garbage has not been previously described but is suspected to vary by poultry industry sector. A survey of representatives from the broiler, turkey, and layer sectors in the United States revealed that many potentially contaminated or infectious items are routinely disposed of in the trash on commercial poultry premises. On-farm garbage management practices, along with trash hauling and disposal practices are thus key components that must be considered to evaluate the risk of commercial poultry becoming infected with HPAI virus.

## Introduction

In past avian influenza (AI) outbreaks in US poultry, evidence of lateral disease spread has been documented *via* transfer of people, vehicles, and shared equipment or visitors between farms (1). Before 2015, however, epidemiological trace-back questionnaires in AI outbreaks on commercial poultry farms in the United States did not specifically investigate garbage management services as a risk factor for disease spread.

Many and likely most commercial poultry operations in the United States use third-party companies to collect and transport trash to off-site disposal locations. Garbage management poses a risk for potential HPAI-virus infection of a commercial poultry flock through a number of pathways. These include: multiple poultry premises (commercial and backyard operations) sharing a common trash collection service provider, sharing a trash collection site (i.e., common dumpster for multiple premises) or disposal site (i.e. landfill). HPAI virus may be carried onto a poultry premises *via* contaminated garbage transport vehicles or drivers, and it is hypothesized that garbage contents within the truck may contain virus-laden trash items. Garbage trucks coming near the barns (within 15 ft) were identified as a significant risk factor in a case–control study in the 2015 United States HPAI H5N2 outbreak. It was shown that egg layer flocks in Nebraska and Iowa that had garbage trucks coming near the barns were 14.7 times more likely to be infected (at the farm level) than flocks that did not have garbage trucks come near the barns (*p* < 0.001) (2). Of note, the frequency with which garbage trucks visited the farms in this study is not known.

To date there are no known studies describing disposal practices used by commercial poultry operations in the United States To more fully evaluate the risk of HPAI infection to commercial poultry *via* garbage management, we initiated a survey of the poultry industry to refine the risk and establish mitigation measures.

## Methods

A convenience sample of veterinarians and other managers in the poultry industry was surveyed between June and August 2016 on standard practices for garbage management on farms that they manage or supervise. A URL link to the survey was distributed to members of the Secure Egg, Turkey, and Broiler Supply working groups *via* email; these groups consisted of industry veterinarians and production managers within major United States poultry producing companies (Appendix S1 in Supplementary Material). The survey was administered using an online polling service.^1^ Participants were surveyed anonymously, minimal opt-in demographic questions (such as company name or job position within the organization) were also included. Some minor differences in the survey wording were used to match common terminology for the commodity (broiler, turkey, or layer) to which it was distributed. In addition, participants were given the option to decline to answer any question within the survey. Respondents were stratified by industry sector (broiler chicken, layer chicken, or turkey) and descriptive statistics were calculated for each. The study was submitted to the University of Minnesota Institutional Review Board and determined to be exempt from review.

## Results

A total of 63 surveys were completed. Respondents represented the turkey (*n* = 15), broiler (*n* = 8), and layer (*n* = 40) commodities. The types of potentially infectious or contaminated material disposed of in the garbage varied by sector of the poultry industry, and many potentially contaminated or infectious materials were reported as routinely disposed of in the trash as listed in Table 1. One or more items classified as a risk (e.g., poultry or wild bird carcasses and items that contacted birds or bird feces) were reported to be disposed of in trash on premises managed by 79.4% of all respondents (layers 75% *n* = 30; broilers 75% *n* = 6; and turkeys 93.3% *n* = 14).

**Table 1:** Survey results of material disposed of in the garbage on premises in the broiler, turkey, and layer industries.^a^

Item	Broiler sector (*n* = 8 respondents)	Turkey sector (*n* = 15 respondents)	Layer sector (*n* = 39 respondents)
Dead wildlife/wild birds	Yes (1/8)	Yes (5/15)	Yes (1/39)
Rodents	Yes (3/8)	Yes (5/15)	Yes (10/39)
Dead poultry or poultry carcasses	No (0/8)	Yes (1/15)	Yes (9/39)
Eggs or egg products^b^	Yes (1/8)	Yes (1/15)	Yes (8/39)
Manure	No (0/8)	No (0/15)	Yes (1/39)
Spilled feed	Yes (2/8)	Yes (8/15)	Yes (7/39)
Disposable chick transport boxes^b^	Yes (4/8)	Yes (4/15)	Yes (24/39)
Used needles/syringes/diagnostic supplies that have contacted birds^b^	Yes (1/8)	Yes (5/15)	Yes (14/39)
Personal protective equipment (boot covers, gloves, coveralls, etc.)	Yes (8/8)	Yes (14/15)	Yes (36/39)
Feathers	No (0/8)	Yes (2/15)	Yes (4/39)
Offal	No (0/8)	No (0/15)	No (0/39)
Equipment or supplies from inside barns^c^	Yes	Yes	Yes (22/39)
Household garbage from farm manager or any other residence^c^	–	Yes	Yes (20/39)
Trash associated with waterfowl hunting^c^	–	–	No (0/39)
Garbage from processing operation^c^	–	–	Yes (23/39)
Lunch room and restroom garbage^c^	–	–	Yes (37/39)

*^a^Yes indicates materials disposed of in the garbage by one or more survey respondents within each industry. In parenthesis, numerator indicates number of survey respondents reporting disposal of item and denominator indicates total number of respondents*.

*^b^Language of selection choice modified in survey distributed to representatives of layer industry*.

*^c^Item only explicitly asked in survey distributed to representatives of layer industry. Yes in the broiler and turkey industries for these items represent at least one write-in response indicating disposal of that item*.

Approximately half of broiler and turkey sector respondents reported that the garbage truck may collect waste from multiple poultry premises before depositing the load at a landfill (43 and 53% respectively), while an additional 48% (*n* = 23) of respondents from all three sectors reported they did not know if the garbage truck route included other poultry premises.

**Figure 1:**
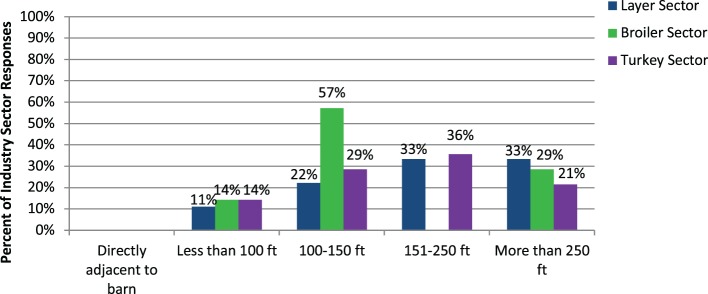
Histogram of frequencies of responses from poultry industry representatives regarding the distance of the dumpster or trash collection point from the nearest poultry barn (layer sector: *n* = 36; broiler sector: *n* = 7; turkey sector: *n* = 14). In the survey of layer industry representatives, it was specified that the nearest poultry barn may be on the same premises or neighboring premises.

The dumpster or garbage collection area may be located at various locations on a premises (reported proximity to the nearest barn of <100 ft (30.48 m) to >250 ft (76.2 m); Figure 1), however only a minority of respondents (*n* = 2; 3.3%) reported sharing a trash collection location between multiple premises. Representatives of all three industry sectors suggest it is common practice for the dumpster or trash collection point to be located at the entrance or perimeter of the farm. This exact distance to the nearest poultry barn may vary; however, this appears to represent a distance of at least 100 ft (30.48 m) to the nearest barn for a majority of respondents.

## Discussion

In our study, respondents identified potential HPAI contaminated or infectious material (i.e., dead wildlife, poultry carcasses, egg shells, and materials that have contacted poultry) that are regularly disposed of in the garbage on their poultry premises. Estimates of HPAI-virus concentrations in chicken and turkey secretions, feces, feathers, and other tissues generally range between 10^3^ and 10^7^ EID_50_ per gram of solid or per milliliter of liquid (3–10), and virus persistence is generally longer at cooler temperatures and in more humid conditions. Virus survival on materials that may be disposed of in the garbage, such as poultry carcasses, feathers, egg shells, egg trays, wood, steel, glass, and personal protective equipment, has been reviewed elsewhere (11–15). Viruses may survive days to weeks or longer depending on environmental conditions. Thus, we suggest the potential for HPAI virus to be present in the garbage and survive in that environment is sufficient to infect a bird should the bird become exposed to that material.

Study participants reported that garbage management contractors used by some turkey and broiler premises visit multiple poultry premises on one route before depositing a load at the landfill; thus, the pathway by which HPAI virus-contaminated garbage from infected premises may be present on the truck when it arrives at the next poultry farm appears to be viable. The types of potentially contaminated trash from non-commercial poultry operations and related industries (e.g., backyard poultry, processing facilities, and live bird markets) are not known, but are likely to include materials similar to those reported in garbage from commercial poultry operations. Poultry carcasses have been reported in the trash of backyard chicken keepers during an exotic Newcastle disease outbreak in California in 2002 (A. Jones, personal communication, September 2017). In the Netherlands, poor waste management practices pertaining to liquid waste (e.g., waste water) and solid waste have been identified as potentially increasing the risk of AI transmission in the neighborhood of infected farms (A. Ssematimba, personal communication, August 2016) (16). A shared dumpster or common trash collection point for multiple poultry premises, while not a common practice in the United States poultry industry, represents an additional site of potential cross-contamination between commercial poultry operations related to garbage management.

Garbage trucks and drivers typically do not contact live poultry while completing contracted duties on poultry premises. Biosecurity recommendations and site-specific biosecurity plans may not stipulate specific biosecurity measures for garbage truck drivers; however, it is recommended in recent updates to the National Poultry Improvement Plan guidance that all visitors and vehicles remain as far from poultry barns as possible (e.g., outside the “Perimeter Buffer Area” or PBA), and for those vehicles which must come near poultry barns, all must be cleaned and disinfected (17). If garbage management activities and pickups occur outside of the PBA, there may be a decreased likelihood of contaminated garbage vehicles, personnel, or virus-laden garbage on the truck contacting farm personnel or equipment which may access the poultry house and expose birds to HPAI virus.

An overwhelming majority of respondents in our survey indicated that they hire a contractor for some or all of their garbage transport needs. Similar to activities of other third-party contractors, cleaning and disinfection of garbage transport vehicles, pickup routing, and landfill practices may be difficult to control and may not be easily influenced by the poultry grower or integrator if using a contractor to haul garbage.

The use of hauling routes that include multiple farms and the use of communal landfills increase the likelihood of contact with infectious garbage. It appears reasonable that garbage within a truck upon arrival to a commercial poultry farm could originate from both commercial and non-commercial (live poultry markets and backyard) poultry operations. In previous outbreaks of HPAI in non-commercial poultry operations, disposal of dead poultry in garbage was noted as a practice which correlated with risk for AI infection. In an evaluation of risk factors for live bird markets in New York, New Jersey, Pennsylvania, and New England, markets that disposed of dead birds and offal in the trash were 2.4 times more likely to have a repeated presence of LPAI H5 and H7 viruses (OR: 2.4; 95% CI, 1.8–3.4) (18). In an analysis of risk factors associated with H5N1 in backyard poultry in Egypt from 2010 to 2012, disposing of dead birds and poultry feces in garbage piles outside was highly correlated with infection in the regression model (*F* = 15.7; *p* < 0.0001) (19). Whether disposing birds in the garbage represented a risk for infection on one’s own premises, or rather is indicative of likelihood for other high-risk practices in these non-commercial operations is not clear. The final destination of the garbage and garbage vehicles, such as to a landfill, also can contribute to the risk of HPAI-virus contamination. Landfills may serve as a potential site for cross-contamination as contracted garbage management services for poultry premises may transport garbage to the same landfill; it has been noted that upon arrival at landfills, garbage hauling vehicles may drive over previously deposited garbage (D. Halvorson, personal communication, June 2016). This risk of vehicle contamination likely increases if landfills are used as an off-site disposal method for infected depopulated flocks, which has been reported in previous LPAI outbreaks (20, 21). Landfills also attract wild birds, including scavenger species such as gulls which are susceptible to HPAI viruses and are a known reservoir of AIVs (22, 23).

This survey used a purposive sampling method focused on recruiting participants with significant experience in the poultry industry and was subsequently limited by small sample size. Members of the surveyed working groups were encouraged to share the survey with others within their companies who might have first-hand knowledge of garbage management practices on poultry farms. Therefore, it is not possible to calculate a reliable response rate for this survey and results may not be generalizable to the entire United States commercial poultry industry. Still, the data are informative for the purpose of risk assessment and serve to illustrate the variations in industry practices and potential differences between poultry sectors that may operate in the same geographic area. As such, we suggest the absence of an affirmative response to a high-risk activity does not definitively indicate it is not occurring, and that further evaluation of the prevalence of such practices on an industry-wide scale may be warranted based on this exploratory survey.

## Conclusion

This exploratory survey identified items in garbage that may contain infectious HPAI virus, some of which may carry high titers of infectious virus. Given that there is potential for HPAI virus to be associated with trash contents and garbage management practices, and taking into account the ease with which virus could be introduced into the poultry house, the potential for a commercial poultry flock becoming infected with HPAI virus due to garbage management during an outbreak should be considered. Further research is needed to determine prevalence of garbage management practices in different production systems and across geographic regions in the United States and producers should develop appropriate mitigation measures in the event of a HPAI outbreak in commercial poultry.

## Ethics Statement

The study was submitted to the University of Minnesota Institutional Review Board and determined to be exempt from review.

## Author Contributions

Survey design and distribution: EW, FC, JU, and EL. Manuscript writing: EW, EL, DH, CC, and MC. Manuscript editing: DH, EL, JU, CC, MC, and FC.

## Conflict of Interest Statement

The authors declare that the research was conducted in the absence of any commercial or financial relationships that could be construed as a potential conflict of interest.

## Footnotes

^1^Qualtrics© 2015 Provo, UT, USA. http://www.qualtrics.com.
