# Supplementary material for: Garbage Management: An Important Risk Factor for HPAI-Virus Infection in Commercial Poultry Flocks
Source: Front Vet Sci. 2018 Jan 26;5:5. doi: 10.3389/fvets.2018.00005 (PMC5790769; doi:10.3389/fvets.2018.00005)
Supplement: Supplementary file 1 [file Data_Sheet_1.PDF]

Supplementary Material

Garbage management: An important risk factor for HPAI-virus infection in commercial poultry flocks

Emily Walz*, Eric Linskens, Jamie Umber, Marie Culhane, David Halvorson, Francesca Contadini, Carol Cardona

*** Correspondence:** Corresponding Author: walzx148@umn.edu

1 Supplementary Data

Survey questions which were distributed using an online polling service are available below. Some minor differences in the survey wording were used to match common terminology for the commodity (broiler, turkey, or layer) to which it was distributed. Wording for turkey-sector survey is not shown but mirrored broiler-sector text.

Garbage management survey: Broiler chickens

Section 1. Garbage content

Company (optional):

____

Which of the following are disposed of in trash/garbage on your premises
(check all that apply)

- ☐ Dead wildlife/wild birds (1)
- ☐ Rodents (2)
- ☐ Mortality or poultry carcasses (3)
- ☐ Eggs or egg products (shells, etc) (4)
- ☐ Manure (5)
- ☐ Spilled feed (6)
- ☐ Disposable chick transport boxes (7)
- ☐ Used needles/syringes/diagnostic supplies that have contacted birds (8)
- ☐ PPE (boot covers, gloves, coveralls, etc) (9)
- ☐ Feathers (10)
- ☐ Offal (11)
- ☐ Other (please describe) (12) ____

Do you put wild bird carcasses in the garbage?

- ○ Yes (1)
- ○ No (2)
- ○ Not sure (3)

Do you ever throw broiler carcasses in your garbage?

- ◯ Never (1)
- ◯ Sometimes (once a week) (2)
- ◯ Often (2-3 times a week) (3)
- ◯ Always (4)

Would you put broiler carcasses in the garbage in the event of an outbreak?

- ◯ Yes (1)
- ◯ Maybe (2)
- ◯ No (3)

Do you put in your garbage other items that may act as fomites? (e.g. used needle/syringes, PPEs,…)

- ◯ Yes (1)
- ◯ Maybe (2)
- ◯ No (3)

Section 2. Garbage movement

How is garbage transported to the landfill?

- ○ Contract garbage hauling company picks up for transportation (1)
- ○ Grower/employee transports the garbage to the landfill (2)
- ○ Other (please describe) (3) ____

Where is the dumpster or trash collection point located on the premise?

- ☐ Container at the entrance to farm (1)
- ☐ Container on the perimeter of farm (2)
- ☐ Shared container utilized by multiple premises (3)
- ☐ Container is located near rendering pickup location (4)

On average, how close is the dumpster/trash collection point to the nearest poultry barn?

- ○ Directly adjacent to barn (1)
- ○ Less than 100ft (2)
- ○ 100-150ft (3)
- ○ 151-250ft (4)
- ○ More than 250ft (5)

Where is the rendering area located in respect to the garbage collection?

- ○ Directly adjacent (1)
- ○ Less than 100ft (2)
- ○ Less than 100-150ft (3)
- ○ More than 150 ft (4)

Does the garbage truck collect waste from multiple poultry premises before depositing the load at a landfill?

- ○ yes (1)
- ○ No (2)
- ○ Not sure/Unknown (3)
- ○ Not applicable- no garbage truck used (4)

How often is your garbage picked up?

- ○ Daily (1)
- ○ Multiple times per week (2)
- ○ Weekly (3)
- ○ Every other week (4)
- ○ Once a month (5)
- ○ Other (please specify) (6) ____

Who is responsible for contracting a garbage pick up/disposal service?

- ○ Integrator (1)
- ○ Grower (2)
- ○ Other (please specify) (3) ____

Are you aware of the presence of other farms on the garbage truck route?

- ○ Yes (1)
- ○ No (2)

In the event of a HPAI outbreak, would it be feasible to suspend garbage collection on the farm for the duration of PMIP?

- ○ Yes (1)
- ○ No (please explain) (2) ____

Garbage management survey- Egg layer chickens

The following questions will ask you to describe the types and frequencies that different items are disposed of in the garbage on your premises.

Which of the following are disposed of in trash/garbage on your premises? (check all that apply)

- ☐ Dead wildlife/ wild birds (1)
- ☐ Rodents (2)
- ☐ Mortality or poultry carcasses (3)
- ☐ Eggs or egg products (shells, etc) (4)
- ☐ Manure (5)
- ☐ Equipment or supplies from inside barns (manure belts, nest pads, etc) (6)
- ☐ Spilled feed (7)
- ☐ Disposable transport boxes/papers (chick boxes, egg flats) (8)
- ☐ Needles/ syringes/ diagnostic supplies that have contacted birds (9)
- ☐ PPE (boot covers, gloves, coveralls, etc) (10)
- ☐ Feathers (11)
- ☐ Offal (12)
- ☐ Household garbage from farm manager residence or any other residence (13)
- ☐ Trash associated with waterfowl hunting (food storage wrap/bags used to store wild duck/goose meat), carcass, feathers, clothing, other) (14)
- ☐ Garbage from processing operation (if applicable) (16)
- ☐ Lunch room and restroom garbage (17)
- ☐ Other (please describe) (15) ____

How often are wild bird carcasses disposed of in your garbage?

- Never (1)
- Infrequently (less than once a week) (5)
- Sometimes (once a week) (2)
- Often (2-3 times a week) (3)
- Always (4)

How often are pullet/layer carcasses disposed of in your garbage?

- Never (1)
- Infrequent/Routinely (eg spent hen disposal) (5)
- Sometimes (once a week) (2)
- Often (2-3 times a week) (3)
- Always (4)

Would you put layer/pullet carcasses in the garbage in the event of an outbreak?

- Yes (1)
- Maybe (2)
- No (3)

Do you put in your garbage other items that may act as infectious disease fomites? (e.g. used needle/syringes, PPEs, ...)

- ○ Never (1)
- ○ Routinely but infrequently (as related to flock management for example chick papers, broken plastic flats, old egg belts) (5)
- ○ Sometimes (once a week) (2)
- ○ Often (2-3 times a week) (3)
- ○ Always (4)

The following questions will ask you to describe the types and frequencies of garbage movement that occur on your premises.

How is garbage transported to the landfill?

- ○ Contract garbage hauling company picks up for transport to landfill (1)
- ○ Grower/employee transports garbage to landfill (2)
- ○ Other (please describe) (3) ____

Where is the dumpster or trash collection point located on the premises? (Check all that apply)

- ☐ Container present at each house (1)
- ☐ Container located at entrance/perimeter of farm (2)
- ☐ Contain on farm but outside Perimeter Buffer Area (PBA) (3)
- ☐ Container located near barns inside PBA (4)
- ☐ Shared container utilized by multiple premises (5)

If the dumpster or trash collection point is shared by multiple premises, where is it located (check all that apply)

- ☐ Container at entrance/perimeter of farm (1)
- ☐ Container on farm but outside Perimeter Buffer Area (PBA) (2)
- ☐ Container located near barns inside PBA on one of the premises (3)
- ☐ Common collection point is outside of perimeters of sharing premises (4)

How is trash collection equipment/dumpster area maintained? (Check all that apply)

- ☐ Pest control is in place to discourage scavengers and flies (1)
- ☐ Trash containers close completely and lids cannot blow open (2)
- ☐ Trash container is maintained so material cannot fall out of bin/dumpster (3)

On average, how close is the dumpster/trash collection point to the nearest poultry barn (on same premise or neighboring premise)?

- ◯ Directly adjacent to barn (1)
- ◯ Less than 100 ft (2)
- ◯ 100-150 ft (3)
- ◯ 151-250 ft (4)
- ◯ More than 250 ft (5)

Where is the rendering area located in respect to the garbage collection point?

- ◯ Directly adjacent (1)
- ◯ Less than 100 ft (2)
- ◯ 100-150 ft (3)
- ◯ More than 150 ft (4)
- ◯ No rendering used (5)

Does the garbage truck collect waste from multiple poultry premises before depositing the load at a landfill?

- Yes (1)
- No (2)
- Unsure/ not known (3)
- Not applicable- no garbage truck used (4)

How often is your garbage picked up or transported to the dump?

- Daily (1)
- Multiple times per week (2)
- Weekly (3)
- Every other week (4)
- Once a month (5)
- Other (please specify) (6) ____

Who is responsible for contracting a garbage pick up/disposal service?

- ◯ Producer/processor (1)
- ◯ Contract producer (2)
- ◯ Pullet farm manager (3)
- ◯ Other (please specify) (4) ____

In the event of a HPAI outbreak, would it be feasible to suspend garbage collection on the farm for the duration of the Pre-Movement Isolation Period (PMIP)?

- ◯ Yes (1)
- ◯ No (please explain why) (2) ____

Optional Demographic Information
Please describe the operations that you are representing in this survey. While this section is optional, it will help us understand if we have a representative picture of the industry

Type of facility - optional (check all that apply)

- ☐ Conventional layer- processing on site (1)
- ☐ Conventional layer- no processing on site (2)
- ☐ Cage free layer (outdoor access)- egg processing on site (5)
- ☐ Cage free layer (no outdoor access)- egg processing on site (7)
- ☐ Cage free layer (outdoor access)- no egg processing on site (8)
- ☐ Cage free layer (no outdoor access)- no egg processing on site (9)
- ☐ Pullet raiser (3)
- ☐ Breeder (4)
- ☐ Other (please describe) (6) ____

Company affiliation (optional)

____

2 Supplementary Figures and Tables

(none)

2.1 Supplementary Figures

(none)
